# Four Pillars in Type 2 Diabetes and Chronic Kidney Disease G4-G5: Mission Impossible—or Not Too Late to Start?

**DOI:** 10.1016/j.xkme.2026.101516

**Published:** 2026-09-07

**Authors:** Masayuki Yamanouchi, Naoki Sawa, Kengo Furuichi, Takashi Wada

**Affiliations:** 1 Nephrology Center, Toranomon Hospital, Tokyo, Japan; 2 Nephrology Center, Toranomon Hospital Kajigaya, Kanagawa, Japan; 3 Department of Nephrology, Kanazawa Medical University School of Medicine, Ishikawa, Japan; 4 Department of Nephrology and Rheumatology, Kanazawa University, Ishikawa, Japan

**Keywords:** Type 2 diabetes, advanced chronic kidney disease, CKD G4-G5, 4-pillar therapy, RAS inhibitors, SGLT2 inhibitors, finerenone, GLP-1 receptor agonists

## Abstract

The 4-pillar therapy—renin-angiotensin system inhibitors, sodium-glucose cotransporter 2 inhibitors, the nonsteroidal mineralocorticoid receptor antagonist finerenone, and glucagon-like peptide 1 receptor agonists—is increasingly applied to improve kidney and cardiovascular outcomes in adults with type 2 diabetes and chronic kidney disease (CKD). However, landmark trials underpinning current recommendations largely enrolled participants down to estimated glomerular filtration rate thresholds of ∼20-30 mL/min/1.73 m^2^, leaving uncertainty in CKD G4-G5. This uncertainty contributes to an implementation gap in advanced CKD, which is as follows: missing pillars are often not initiated, therapies are interrupted during clinical complications, and they are not reinitiated once acute triggers resolve despite high absolute kidney and cardiovascular risk. This narrative review summarizes what current evidence does—and does not—support for initiating, continuing, temporarily withholding, discontinuing, and reinitiating each pillar in type 2 diabetes and CKD G4-G5. We integrate posthoc and subgroup analyses of landmark trials, meta-analyses, and pooled analyses, including low-estimated glomerular filtration rate strata and observational real-world data. The main practical message is that current evidence generally supports a monitoring-based approach to 4-pillar therapy in CKD G4, whereas decisions in CKD G5 remain more individualized and explicitly extrapolative. For clinicians, the key priority is to identify missing pillars, avoid unnecessary permanent discontinuation, and use structured reinitiation when clinically appropriate.

Adults with type 2 diabetes and advanced chronic kidney disease (CKD G4-G5; estimated glomerular filtration rate [eGFR] < 30 mL/min/1.73 m^2^) represent one of the highest risk populations in kidney and cardiovascular medicine.^1^ As eGFR declines below 30 mL/min/1.73 m^2^, absolute risks of kidney failure, heart failure hospitalization, atherosclerotic cardiovascular events, and death increase substantially.^1^ At the same time, therapeutic decisions become constrained by competing priorities, including hemodynamic intolerance, electrolyte disturbances, frailty, polypharmacy, acute intercurrent illness, and limitations of laboratory monitoring in routine care.^1^ This combination creates an implementation paradox in advanced CKD, which is as follows: the stage with potentially the greatest absolute benefit from risk-reducing therapy is also the stage in which therapies are least likely to be initiated and most likely to be withheld, interrupted, or not reinitiated.

A 4-pillar approach—renin-angiotensin system (RAS) inhibitors, sodium-glucose cotransporter 2 (SGLT2) inhibitors, the nonsteroidal mineralocorticoid receptor antagonist (MRA) finerenone, and glucagon-like peptide 1 (GLP-1) receptor agonists—is increasingly proposed to reduce kidney and cardiovascular events in adults with type 2 diabetes and CKD.^2^, ^3^, ^4^, ^5^ This framework is supported by large randomized trials demonstrating kidney and/or cardiovascular benefits for each class. However, pivotal trials largely enrolled participants down to eGFR thresholds of ∼20-30 mL/min/1.73 m^2^, resulting in fewer participants and fewer events in the most advanced strata and leaving uncertainty regarding initiation and continuation in CKD G4-G5 (Fig 1). Because dedicated randomized evidence in CKD G4-G5 remains limited, clinically useful synthesis requires integrating complementary evidence streams. In this narrative review, we integrate the following aspects: (1) posthoc and subgroup analyses of landmark trials, (2) meta-analyses including low-eGFR strata, and (3) real-world data, including causal inference–oriened observational analyses. We frame the evidence around pragmatic clinical decisions in advanced CKD—when to initiate missing pillars, when to continue, and when to temporarily withhold, discontinue, or reinitiate therapy—highlighting what is supported by current data, where uncertainties remain, and priorities for future research.

**Figure 1:**
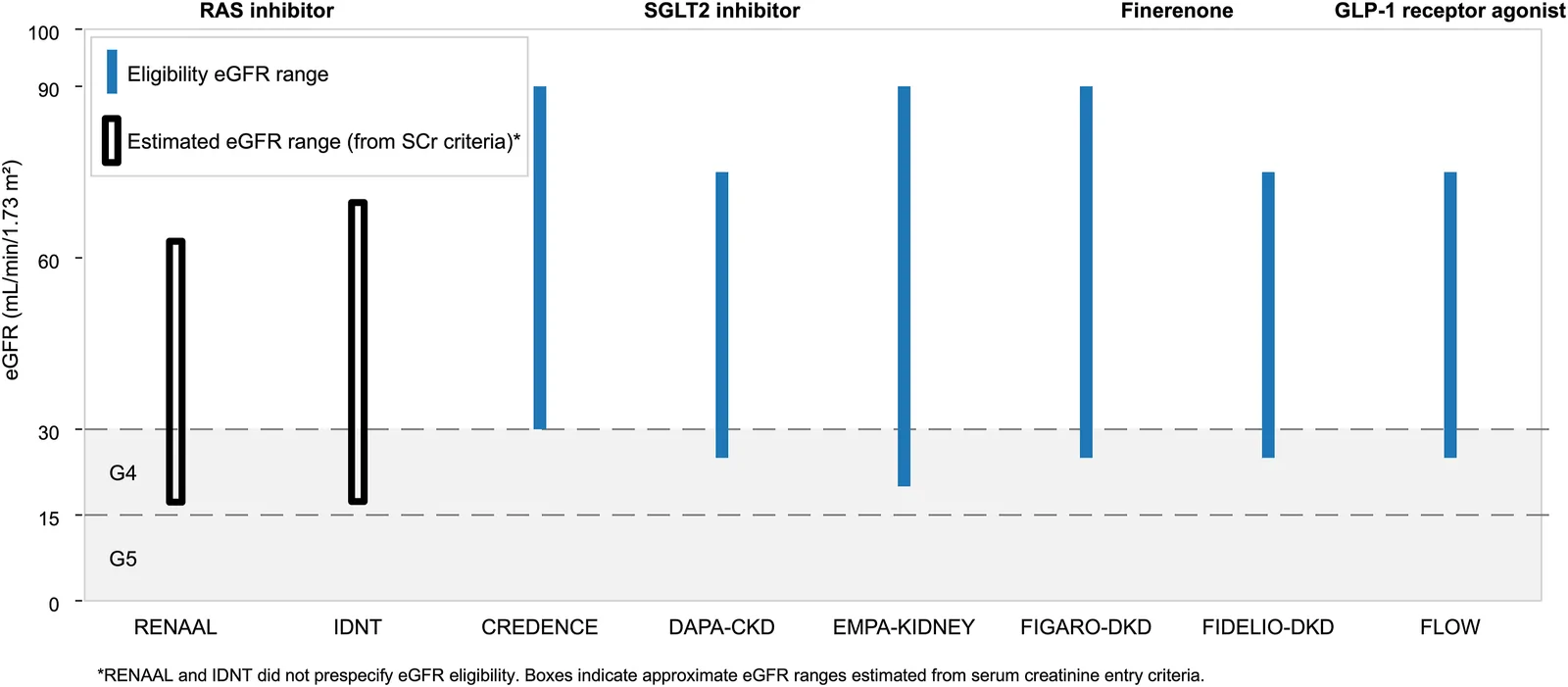
Eligibility eGFR ranges across landmark trials informing 4-pillar therapy in type 2 diabetes and CKD. Vertical bars indicate the approximate eGFR ranges allowed by the entry criteria of landmark trials supporting each therapeutic pillar. For RENAAL and IDNT, eGFR eligibility was not prespecified and estimated from serum creatinine–based criteria. The figure illustrates eligibility boundaries, not treatment effect size or strength of recommendation, and highlights the limited direct randomized evidence in CKD G4-G5. Abbreviations: CKD, chronic kidney disease; CREDENCE, canagliflozin and renal events in diabetes with established nephropathy clinical evaluation; DAPA-CKD, dapagliflozin and prevention of adverse outcomes in chronic kidney disease; eGFR, estimated glomerular filtration rate; EMPA-KIDNEY, Study of Heart and Kidney Protection With Empagliflozin; FIDELIO-DKD, finerenone in reducing kidney failure and disease progression in diabetic kidney disease; FIGARO-DKD, finerenone in reducing cardiovascular mortality and morbidity in diabetic kidney disease; FLOW, Evaluate Renal Function With Semaglutide Once Weekly; GLP-1, glucagon-like peptide 1; IDNT, Irbesartan Diabetic Nephropathy Trial; RAS, renin-angiotensin system; RENAAL, reduction of endpoints in NIDDM with the angiotensin II antagonist losartan; Scr, serum creatinine; SGLT2, sodium-glucose cotransporter 2.

## Pillar 1: RAS Inhibitors

### Guideline Positioning and the Evidence Gap in CKD G4-G5

RAS inhibition remains foundational for albuminuric CKD in type 2 diabetes, supported by established benefits on albuminuria reduction and kidney disease progression from classic diabetic nephropathy trials (eg, Reduction of Endpoints in NIDDM with the Angiotensin II Antagonist Losartan and Irbesartan Diabetic Nephropathy Trial).^6^^,^^7^ However, these trials were not designed to specifically test initiation in CKD G4-G5, and precision is limited in the most advanced strata. In practice, contemporary guidance emphasizes use at the maximum-tolerated dose with appropriate monitoring and discourages stopping therapy solely because eGFR is low in the absence of clinically meaningful intolerance.^2^^,^^4^^,^^5^

### Posthoc/Subgroup Evidence Relevant to CKD G4-G5

A pivotal randomized trial directly addressing a central G4-G5 dilemma is the STOP-ACEi (Multicentre Randomized Controlled Trial of Angiotensin-Converting Enzyme Inhibitor/Angiotensin Receptor Blocker Withdrawal in Advanced Renal Disease) trial, which compared discontinuation versus continuation of angiotensin-converting enzyme inhibitors or angiotensin II receptor blockers in advanced CKD (not restricted to diabetic CKD).^8^ At 3 years, the least-squares mean eGFR did not differ meaningfully between groups (12.6 vs 13.3 mL/min/1.73 m^2^; difference −0.7 [−2.5 to 1.0]; *P* = 0.42), and the results did not support routine withdrawal as a default strategy to improve kidney function trajectory.^8^ Initiation of kidney replacement therapy occurred in 62% versus 56%, respectively (hazard ratio [HR] 1.28 [0.99-1.65]); cardiovascular events (108 vs 88) and deaths (20 vs 22) were similar.^8^ Thus, discontinuation should not be a reflexive response to low eGFR alone.^8^

### Meta-analyses and Pooled Analyses Including Low-eGFR Strata

Meta-analyses synthesizing low-eGFR populations provide complementary evidence in which individual trials have limited precision. In CKD, including advanced CKD strata, pooled estimates generally suggest that discontinuation (vs continuation) of RAS inhibitors is associated with worse outcomes, although heterogeneity and confounding based on indication remain limitations. In a systematic review and meta-analysis, discontinuation was associated with higher risks of all-cause mortality (HR 1.42 [1.23-1.63]) and cardiovascular events (HR 1.25 [1.17-1.32]); the pooled association with kidney failure with replacement therapy was also higher (HR 1.23 [1.02-1.49]), although estimates for kidney outcomes were more sensitive to study mix and follow-up.^9^ In subgroup analyses, the cardiovascular event risk associated with discontinuation remained elevated in eGFR < 30 mL/min/1.73 m^2^ (HR 1.31 [1.26-1.37]), whereas the association with kidney failure with replacement therapy in eGFR < 30 mL/min/1.73 m^2^ was not statistically significant (HR 1.09 [0.88-1.36]).^9^

Complementary evidence also addresses initiation at low eGFR, which is as follows: in a retrospective individual participant-level meta-analysis of 18 randomized trials (mean baseline eGFR 22.2 mL/min/1.73 m^2^), initiation of angiotensin-converting enzyme inhibitors/angiotensin II receptor blockers (vs non–angiotensin-converting enzyme inhibitor/angiotensin II receptor blocker comparators) was associated with a lower risk of kidney failure with replacement therapy (adjusted HR 0.66 [0.55-0.79]), without a clear reduction in death (HR 0.86 [0.58-1.28]).^10^

### Real-World Data, Including Causal Inference–Oriented Observational Analyses

Across health systems, discontinuation of RAS inhibitors in advanced CKD is common, often triggered by hyperkalemia, acute kidney injury, hypotension, or perceived proximity to dialysis. Large cohort studies in type 2 diabetes and advanced CKD suggest that discontinuation is associated with higher risks of adverse outcomes, including death and cardiovascular events, although confounding by indication remains a central limitation.^11^ In an observational target-trial emulation study after incident hyperkalemia (K ≥ 5.5 mEq/L), discontinuation was associated with a higher hazard of all-cause mortality (adjusted HR 1.16 [1.02-1.33]), whereas the composite kidney outcome was similar (adjusted HR 1.01 [0.81-1.26]); severe hyperkalemia (K ≥ 6.5 mEq/L) occurred less frequently with discontinuation (adjusted HR 0.83 [0.69-0.99]), illustrating the tradeoff between hyperkalemia control and survival.^12^

More recently, a target-trial emulation study among new users of RAS inhibitors who experienced an acute decline in eGFR of >15% within 90 days after initiation found that discontinuation (vs continuation) was associated with higher risks of all-cause mortality (HR 1.23 [1.07-1.41]) and kidney failure with replacement therapy (HR 1.74 [1.28-2.38]), without a significant increase in major adverse cardiovascular events or acute kidney injury.^13^ Reinitiation is clinically important in CKD (including advanced CKD), because temporary interruptions often become permanent pillar loss. In a target-trial emulation study among patients who discontinued RAS inhibitors, restarting within 1 year (vs not restarting), was associated with lower hazards of a composite kidney outcome (HR 0.85 [0.78-0.93]) and all-cause death (HR 0.70 [0.61-0.80]), with no clear increase in hyperkalemia (HR 1.11 [0.96-1.27]).^14^ Taken together, these observational and target-trial emulation studies argue against reflex discontinuation and support reevaluation for reinitiation where clinically feasible, although several were not restricted to adults with type 2 diabetes and CKD G4-G5, and all remain vulnerable to residual confounding, treatment-selection bias, and incomplete ascertainment of the reasons for treatment interruption.

### Practical Decision Points in CKD G4-G5: Initiate/Continue/Withhold/Discontinue/Reinitiate

Table 1^1^^,^^2^^,^^4^^,^^8^, ^9^, ^10^, ^11^, ^12^, ^13^, ^14^, ^15^, ^16^, ^17^, ^18^, ^19^, ^20^, ^21^, ^22^, ^23^, ^24^, ^25^, ^26^, ^27^, ^28^, ^29^, ^30^, ^31^, ^32^, ^33^, ^34^, ^35^, ^36^, ^37^, ^38^, ^39^, ^40^, ^41^ displays the practical decision points in CKD G4-G5.

**Table 1:** Guideline Positioning, Evidence Boundary, Practical Use, and Key Limitations of 4-Pillar Therapy in Type 2 Diabetes and CKD G4-G5

Pillar	Guideline Positioning	Evidence Boundary in Advanced CKD	Guideline Support^a^	Practical Use in CKD G4-G5^b^	Key Safety/Implementation Issue	Evidentiary Role/Main Limitation
RAS inhibitors (ACEi/ARB)	Foundational therapy for albuminuric CKD in type 2 diabetes; guidelines generally support use at the maximally tolerated dose and do not recommend routine withdrawal solely because eGFR is low.^2^^,^^4^^,^^5^	No single randomized eGFR “floor” defines use. Evidence is stronger for continuation and reinitiation than for de novo initiation in CKD G5.^8^, ^9^, ^10^	Strong guideline support for albuminuric CKD, although direct randomized evidence specifically focused on de novo initiation in CKD G4-G5 is limited.^2^^,^^4^^,^^5^	CKD G4**:** initiate or continue if albuminuria, blood pressure, potassium, and creatinine trajectory are acceptable and monitoring is feasible. Avoid reflex discontinuation for low eGFR alone. CKD G5**:** individualized; continuation is often more defensible than de novo initiation. Temporary withholding may be appropriate during true AKI, marked hypotension, or acute dehydrating illness, but reinitiation should be considered once the trigger resolves.	Hyperkalemia, hypotension, and creatinine rise may drive avoidable discontinuation. Continuation strategies should include potassium mitigation, diuretic review, correction of reversible contributors, temporary holds rather than reflex permanent discontinuation, and planned reinitiation after recovery.	Foundational class with longstanding kidney benefit in albuminuric diabetic CKD. In advanced CKD, major support comes from continuation/discontinuation studies and target-trial emulations. Observational estimates remain vulnerable to confounding by indication, frailty, illness severity, and monitoring intensity.^6^, ^7^, ^8^, ^9^, ^10^, ^11^, ^12^, ^13^, ^14^
SGLT2 inhibitors	Foundational therapy for kidney and cardiovascular protection in type 2 diabetes and CKD; low-eGFR labeling concerns often reflect reduced glycemic efficacy rather than loss of cardiorenal benefit^2^^,^^4^^,^^5^	Randomized trial evidence extends down to eGFR 20-30 mL/min/1.73 m^2^ depending on the trial. Direct evidence in CKD G5 remains limited, and dialysis evidence is still emerging; ongoing randomized evaluation includes the Renal Lifecycle trial.^15^, ^16^, ^17^, ^18^, ^19^, ^20^	Strong guideline support within trial-supported eGFR ranges, but support becomes more extrapolative in CKD G5 and dialysis populations.^2^^,^^4^^,^^5^	CKD G4**:** initiate when within an evidence-supported range and continue if clinically stable; do not stop automatically when eGFR falls. CKD G5: individualized and extrapolative for de novo initiation; continuation may be reasonable in selected patients already receiving therapy. Temporary withholding is appropriate during acute illness with poor intake, dehydration, perioperative periods, or suspected ketoacidosis, with reinitiation after recovery if previously tolerated.	Volume depletion, poor oral intake, ketoacidosis risk during acute illness, and perioperative interruption. Clear sick-day rules and explicit restart plans are critical to prevent permanent pillar loss.	One of the strongest evidence bases near the low-eGFR boundary. Subgroup analyses and meta-analyses support preserved benefit in CKD G4, but CKD G5 and dialysis data remain sparse and are partly observational or investigational. Posthoc and subgroup analyses are not causal proof at the extreme low-eGFR end.^15^, ^16^, ^17^, ^18^, ^19^, ^20^, ^21^, ^22^, ^23^, ^24^, ^25^
Finerenone (nonsteroidal MRA)	Add-on therapy for persistent albuminuria despite optimized standard care, usually on background RAS inhibition and increasingly with SGLT2 inhibition when feasible.^2^^,^^4^^,^^5^	Randomized outcome-trial evidence supports initiation at eGFR ≥ 25 mL/min/1.73 m^2^. CKD G4 subgroup data exist, but de novo initiation in CKD G5 remains an evidence gap; a small observational study suggested possible implementation feasibility below this threshold, but the evidence remains hypothesis generating only.^26^, ^27^, ^28^, ^29^, ^30^	Guideline-supported add-on therapy within the trial/label boundary, but support becomes more extrapolative below eGFR 25 mL/min/1.73 m^2^ because direct evidence is sparse.^2^^,^^4^^,^^5^	CKD G4: consider initiation for persistent albuminuria if potassium is acceptable and monitoring is reliable; continuation should be guided mainly by potassium, not by eGFR alone. CKD G5: individualized and explicitly extrapolative; continuation after prior initiation may be more defensible than de novo initiation. Temporary withholding may be appropriate for hyperkalemia above a prespecified threshold or during acute illness with clinically meaningful hyperkalemia or true AKI; reinitiation may be considered after potassium normalization and correction of reversible contributors.	Hyperkalemia is the dominant implementation issue and requires structured potassium monitoring, mitigation of reversible contributors, and a lower threshold for temporary holding than for permanent discontinuation.	Evidence in CKD G4 is supportive but still limited, especially for hard kidney endpoints. By contrast, steroidal MRAs have less direct outcome-trial support for adults with type 2 diabetes and CKD than finerenone and greater concerns regarding hyperkalemia and other adverse effects. Real-world finerenone studies below the trial threshold remain hypothesis generating rather than definitive.^26^, ^27^, ^28^, ^29^, ^30^, ^31^, ^32^, ^33^
GLP-1 receptor agonists	Add-on therapy for residual cardiovascular risk, obesity, or inadequate glycemic control, especially when additional risk reduction is needed beyond foundational therapy.^2^^,^^4^^,^^5^	Dedicated kidney-outcome evidence now extends to eGFR ≥ 25 mL/min/1.73 m^2^. Below this range, especially in CKD G5, initiation is extrapolative.^34^, ^35^, ^36^, ^37^, ^38^	Guideline-supported add-on therapy with kidney-outcome support strengthened by FLOW, although certainty declines in CKD G5 because direct randomized evidence is lacking.^2^^,^^4^^,^^5^	CKD G4: consider when nutritional resilience, hydration, and follow-up capacity are adequate; continue if tolerated. CKD G5: individualized and cautious; avoid reflexive use in frail, catabolic, or dehydration-prone patients. Temporary withholding is appropriate during vomiting, diarrhea, poor intake, dehydration, or perioperative periods; reinitiation should be slow and selective after recovery.	Gastrointestinal intolerance, anorexia, weight loss, dehydration, and downstream frailty/sarcopenia risk. Monitoring should include weight, intake, functional trajectory, and volume status, not just glucose indices.	The evidence base is growing and now includes a dedicated kidney-outcome trial, but advanced CKD inference is still less secure than for RAS or SGLT2 inhibitors. Observational studies in advanced CKD are especially vulnerable to residual confounding based on frailty, nutritional status, and treatment selection.^34^, ^35^, ^36^, ^37^, ^38^, ^39^, ^40^, ^41^

*Note:* “Practical use” distinguishes temporary withholding from permanent discontinuation. Across all pillars, temporary interruption should be paired with reassessment and a plan for reinitiation when clinically appropriate. Across all pillars, observational studies and target-trial emulations remain susceptible to residual confounding, including confounding based on frailty, illness severity, treatment selection, competing risk, and monitoring intensity.

Abbreviations: ACEi, angiotensin-converting enzyme inhibitor; ADA, The American Diabetes Association; AKI, acute kidney injury; ARB, angiotensin II receptor blocker; CKD, chronic kidney disease; eGFR, estimated glomerular filtration rate; FLOW, evaluate renal function with semaglutide once weekly; GLP-1, glucagon-like peptide 1; KDIGO, Kidney Disease: Improving Global Outcomes; MRA, mineralocorticoid receptor antagonist; RAS, renin-angiotensin system; SGLT2, sodium-glucose cotransporter 2.

a Formal recommendation-grading systems differ across KDIGO, ADA/KDIGO consensus, and ADA documents. This column therefore summarizes the level of guideline support rather than implying direct equivalence of grading systems.^2^^,^^4^^,^^5^

b In CKD G5, many treatment decisions remain individualized and explicitly extrapolative, because direct randomized evidence is limited or absent.^1^^,^^2^^,^^4^^,^^5^

## Pillar 2: SGLT2 Inhibitors

### Guideline Positioning and the Evidence Gap in CKD G4-G5

Landmark SGLT2 inhibitor trials enrolled participants down to eGFR thresholds of 30 mL/min/1.73 m^2^ in CREDENCE (Canagliflozin and Renal Events in Diabetes with Established Nephropathy Clinical Evaluation), 25 mL/min/1.73 m^2^ in DAPA-CKD (Dapagliflozin and Prevention of Adverse Outcomes in Chronic Kidney Disease), and 20 mL/min/1.73 m^2^ in EMPA-KIDNEY (Study of Heart and Kidney Protection with Empagliflozin).^15^, ^16^, ^17^ Accordingly, direct randomized evidence in the lowest eGFR strata is less precise. Importantly, labeling cautions at low eGFR often reflect limited enrollment and reduced glucose-lowering efficacy rather than evidence of harm or lack of kidney and cardiovascular benefits.^2^^,^^5^ Direct randomized evidence remains limited beyond the main trial boundaries, particularly in patients with eGFR < 25 mL/min/1.73 m^2^, with even less direct evidence in those requiring dialysis or kidney transplant recipients. However, ongoing studies such as the Renal Lifecycle trial may help address this evidence gap.^18^

### Posthoc/Subgroup Evidence Relevant to CKD G4-G5

In a posthoc analysis of CREDENCE focusing on participants with eGFR < 30 mL/min/1.73 m^2^ at treatment initiation (mean eGFR 26.5 mL/min/1.73 m^2^), canagliflozin slowed the mean eGFR decline from weeks 3-130 compared with placebo (−1.30 vs −3.83 mL/min/1.73 m^2^ per year) and reduced albuminuria, with no clear excess of major safety concerns in this small subgroup.^22^

In DAPA-CKD, a prespecified analysis in CKD G4 (eGFR < 30 mL/min/1.73 m^2^) showed an eGFR slope decline of −2.15 versus −3.38 mL/min/1.73 m^2^ per year with dapagliflozin versus placebo (*P* = 0.005) and effects on clinical endpoints consistent within the overall trial, without evidence of increased risks.^23^

In EMPA-KIDNEY, empagliflozin reduced the primary composite of kidney disease progression or cardiovascular death (HR 0.72 [0.64-0.82]), with consistency across eGFR subgroups, including <30 mL/min/1.73 m^2^.^17^ A prespecified secondary analysis focusing on progression metrics showed an acute dip of ∼2.12 mL/min/1.73 m^2^ in the first 2 months, and a halving of the chronic eGFR slope from −2.75 to −1.37 mL/min/1.73 m^2^ per year overall.^42^

Collectively, although confidence intervals widen near the lowest eGFR strata, available trial and subgroup evidence does not suggest loss of kidney benefit or emergence of new safety signals specific to CKD G4.^15^, ^16^, ^17^^,^^21^, ^22^, ^23^

### Meta-analyses and Pooled Analyses Including Low-eGFR Strata

To improve precision at low eGFR, meta-analyses pooling trial data provide an important complementary evidence stream. The SMART-C (SGLT2 Inhibitor Meta-Analysis Cardio-Renal Trialists’ Consortium) consortium meta-analysis across 10 randomized trials (70,361 participants) reported a reduced risk of CKD progression overall (HR 0.62 [0.57-0.68]), with consistent benefit across baseline eGFR categories, including eGFR < 30 mL/min/1.73 m^2^ (HR 0.71 [0.61-0.83]), and reduced kidney failure (HR 0.66 [0.58-0.75]).^19^

In a pooled analysis of placebo-controlled trials across cardiovascular-kidney-metabolic conditions, 1,392 of 26,946 (5.2%) participants experienced time-updated eGFR deterioration to eGFR < 25 mL/min/1.73 m^2^ during follow-up. Severe eGFR deterioration was independently associated with an increased risk of subsequent cardiovascular outcomes and mortality; however, the beneficial impact of SGLT2 inhibitor treatment on cardiovascular outcomes and all-cause mortality was maintained irrespective of eGFR deterioration (interaction *P* > 0.1 for all outcomes).^20^ Patients with eGFR deterioration were more likely to permanently discontinue treatment after deterioration (19% vs 12%), underscoring implementation attrition in this setting.^20^

### Real-World Data, Including Causal Inference–Oriented Observational Analyses

In CKD G5, randomized evidence remains limited; therefore, clinically informative data largely come from observational analyses. In a Taiwanese nationwide emulated target trial in type 2 diabetes and CKD G5, SGLT2 inhibitor use (vs no SGLT2 inhibitor use) was associated with a lower risk of dialysis initiation (HR 0.34 [0.27-0.43]) and lower risks of several adverse outcomes, with no difference in all-cause mortality.^24^

Regarding the common decision to stop therapy after eGFR decreases below 30, a retrospective cohort study reported that continuation (vs discontinuation) after eGFR ≤ 30 mL/min/1.73 m^2^ was associated with lower risks of major adverse cardiovascular endpoints (HR 0.65 [0.43-0.99]), ≥50% eGFR decline (subdistribution HR 0.58 [0.42-0.81]), and all-cause hospitalization (HR 0.77 [0.64-0.94]) without clear safety imbalances.^25^ As with observational analyses across the other pillars, these estimates remain vulnerable to residual confounding, particularly based on frailty, illness severity, treatment selection, competing risk, and monitoring intensity, especially in CKD G5 in which treatment decisions are highly selective.

### Clinical Decision Points in CKD G4-G5: Initiate/Continue/Withhold/Discontinue/Reinitiate

Table 1^1^^,^^2^^,^^4^^,^^8^, ^9^, ^10^, ^11^, ^12^, ^13^, ^14^, ^15^, ^16^, ^17^, ^18^, ^19^, ^20^, ^21^, ^22^, ^23^, ^24^, ^25^, ^26^, ^27^, ^28^, ^29^, ^30^, ^31^, ^32^, ^33^, ^34^, ^35^, ^36^, ^37^, ^38^, ^39^, ^40^, ^41^ displays the clinical decision points in CKD G4-G5.

## Pillar 3: Finerenone (Nonsteroidal MRA)

### Guideline Positioning and the Evidence Gap in CKD G4-G5

Finerenone is recommended as an add-on therapy for adults with type 2 diabetes and CKD with persistent albuminuria despite optimized standard of care, typically on a background of RAS inhibition (and increasingly SGLT2 inhibition).^2^^,^^4^^,^^5^ Pivotal outcome trials enrolled participants down to eGFR ≥ 25 mL/min/1.73 m^2^, and prescribing information emphasizes initiation above this threshold with structured potassium monitoring and dose adjustment.^26^^,^^27^ Thus, direct randomized evidence is limited near the lower eligibility boundary and absent for de novo initiation in CKD G5.^26^^,^^27^ By contrast, steroidal MRAs have less direct outcome-trial support in adults with type 2 diabetes and CKD than finerenone, and their use in this setting is more often constrained by hyperkalemia and other adverse effects.^31^ Accordingly, the present review focuses on finerenone as the nonsteroidal MRA with dedicated kidney and cardiovascular outcome evidence in type 2 diabetes and CKD.

### Posthoc/Subgroup Evidence Relevant to CKD G4-G5

In FIDELIO-DKD (finerenone in reducing kidney failure and disease progression in diabetic kidney disease), finerenone reduced the kidney composite outcome versus placebo, with hyperkalemia as the dominant safety signal.^26^ FIGARO-DKD (finerenone in reducing cardiovascular mortality and morbidity in diabetic kidney disease) demonstrated cardiovascular benefit across a broader CKD spectrum (eGFR > 25 mL/min/1.73 m^2^).^27^ Across both trials, subgroup analyses based on baseline eGFR did not suggest a qualitative loss of benefit down to the eligibility boundary, although estimates became imprecise near the lower limit.

### Meta-analyses and Pooled Analyses Including Low-eGFR Strata

The prespecified FIDELITY (Finerenone in Chronic Kidney Disease and Type 2 Diabetes: Combined FIDELIO-DKD and FIGARO-DKD Trial Programme Analysis) pooled analysis (FIDELIO-DKD + FIGARO-DKD; >13,000 participants) improved precision and reported reductions in both the kidney composite (HR 0.77 [0.67-0.88]) and the cardiovascular composite (HR 0.86 [0.78-0.95]) outcomes, with the expected increase in hyperkalemia risk under protocolized monitoring.^28^

A dedicated CKD G4 analysis (eGFR < 30 mL/min/1.73 m^2^; ∼7% of the FIDELITY pooled cohort) directly addresses the G4 evidence gap as follows: the cardiovascular composite was directionally favorable but imprecise (HR 0.78 [0.57-1.07]); hyperkalemia was more common (26% vs 13%), whereas permanent discontinuation owing to hyperkalemia remained uncommon (3% vs 2%) under trial monitoring.^29^

Importantly, for kidney time-to-event endpoints in CKD G4, nonproportional hazards were observed, making a single HR over the entire follow-up less stable to interpret in this subgroup.^29^ By contrast, mechanistically aligned intermediate signals were robust, which was as follows: finerenone reduced the urinary albumin-creatinine ratio by 31% at month 4 versus placebo (least-squares mean ratio 0.69 [0.63-0.77]; *P* < 0.001), with durability through 24 months, and significantly attenuated the chronic eGFR slope from month 4 to end of treatment (−1.8 vs −3.2 mL/min/1.73 m^2^ per year; least-squares mean difference 1.39 [0.48-2.30]; *P* = 0.04), whereas the total eGFR slope from baseline to end of treatment was numerically but not significantly improved (−0.7 vs −1.6 mL/min/1.73 m^2^ per year; least-squares mean difference 0.84 [0.02-1.67]; *P* = 0.22).^29^ CKD G5 remains a clear evidence gap.

### Real-World Data, Including Causal Inference–Oriented Observational Analyses

Real-world evidence for finerenone is increasing, but high-quality causal inference studies specifically evaluating de novo initiation in CKD G5 remain very limited. Available reports predominantly reflect CKD G3-G4 populations near trial eligibility boundaries and describe albuminuria responses, eGFR trajectories, and potassium-related safety under routine monitoring, including some patients with type 2 diabetes and CKD with baseline eGFR < 25 mL/min/1.73 m^2^.^32^ A small retrospective single-center study in patients with type 2 diabetes and CKD with baseline eGFR < 25 mL/min/1.73 m^2^ reported attenuation of eGFR decline after finerenone initiation, but the sample size was very small and the findings should be interpreted as hypothesis generating rather than confirmatory.^30^ A key limitation is that real-world estimates remain susceptible to residual confounding, particularly based on frailty, illness severity, treatment selection at very low eGFR, competing risk, and monitoring intensity, as well as outcome misclassification. These limitations should be stated explicitly, especially when interpreting apparent benefit near or below trial eligibility boundaries and when extrapolating to CKD G5.

### Clinical Decision Points in CKD G4-G5: Initiate/Continue/Withhold/Discontinue/Reinitiate

Table 1^1^^,^^2^^,^^4^^,^^8^, ^9^, ^10^, ^11^, ^12^, ^13^, ^14^, ^15^, ^16^, ^17^, ^18^, ^19^, ^20^, ^21^, ^22^, ^23^, ^24^, ^25^, ^26^, ^27^, ^28^, ^29^, ^30^, ^31^, ^32^, ^33^, ^34^, ^35^, ^36^, ^37^, ^38^, ^39^, ^40^, ^41^ displays the clinical decision points in CKD G4-G5.

As a hypothesis-generating implementation signal from a heart failure population (rather than advanced CKD), a blinded ∼30-day withdrawal analysis from FINEARTS-HF (Finerenone Trial to Investigate Efficacy and Safety Superior to Placebo in Patients With Heart Failure) suggested a higher incidence of cardiovascular serious adverse events or deaths after study-drug withdrawal among participants previously assigned to finerenone (reported HR 2.80 [1.44-5.45]); this should be interpreted explicitly as non-CKD, short-term withdrawal evidence rather than as a CKD G4-G5 algorithm.^33^

## Pillar 4: GLP-1 Receptor Agonists

### Guideline Positioning and the Evidence Gap in CKD G4-G5

GLP-1 receptor agonists have evolved from glucose-lowering agents to therapies with established cardiovascular benefit and, more recently, dedicated kidney-outcome evidence.^4^^,^^5^ In adults with type 2 diabetes and CKD, major guidelines position GLP-1 receptor agonists as an add-on therapy on top of foundational RAS inhibition and SGLT2 inhibition—particularly when residual cardiovascular risk, obesity, or suboptimal glycemic control persists.^2^^,^^4^^,^^5^ In contrast to other pillars, implementation in advanced CKD is often constrained less by an eGFR-based stopping rule (for several agents, dose adjustment is not required) and more by tolerability and nutritional/volume vulnerability (nausea, anorexia, weight loss, and dehydration), which can be amplified in CKD G4-G5. Randomized kidney-outcome evidence is now strengthened down to eGFR ≥ 25 mL/min/1.73 m^2^ (FLOW [evaluate renal function with semaglutide once weekly]),^34^ whereas de novo initiation in CKD G5 remains an evidence gap.

### Posthoc/Subgroup Evidence Relevant to CKD G4-G5

An exploratory analysis of AWARD-7 (assessment of weekly administration of LY2189265 in diabetes-7) (CKD G3-G4) reported that dulaglutide 1.5 mg weekly was associated with a lower risk of ≥40% eGFR decline or kidney failure with replacement therapy versus insulin glargine over 1 year (HR 0.45 [0.20-0.97]), with a stronger signal among participants with macroalbuminuria (HR 0.25 [0.10-0.68]).^35^^,^^36^

In FLOW (eGFR 25-75 mL/min/1.73 m^2^; urinary albumin-creatinine ratio 300-5,000), semaglutide reduced the primary kidney composite outcome (HR 0.76 [0.66-0.88]) and slowed the mean annual eGFR slope by 1.16 mL/min/1.73 m^2^ per year versus placebo.^34^ Prespecified baseline eGFR subgroup analyses did not show clear heterogeneity based on baseline eGFR, although precision was inherently lower near the eligibility boundary.^34^ In CKD G4-G5, potential benefits must be interpreted alongside tolerability, because gastrointestinal adverse effects can lead to reduced intake and downstream clinical instability in vulnerable patients. Across trials, the dominant safety constraint is gastrointestinal intolerance leading to dose reduction or discontinuation, whereas hypoglycemia is uncommon unless combined with insulin. In advanced CKD, monitoring should explicitly include weight trajectory, appetite/oral intake, volume status, and frailty/sarcopenia risk, because even expected nausea or anorexia can have outsized consequences.

### Meta-analyses and Pooled Analyses Including Low-eGFR Strata

Earlier trial-level meta-analyses of GLP-1 receptor agonist cardiovascular outcome trials suggested kidney benefit signals, but kidney composite definitions varied and were often driven by albuminuria components rather than hard kidney endpoints.^37^

A more recent meta-analysis incorporating FLOW reported reductions in a composite kidney outcome (HR 0.82 [0.73-0.93]) and kidney failure (HR 0.84 [0.72-0.99]) among participants with type 2 diabetes, alongside cardiovascular benefit (major adverse cardiovascular endpoints HR 0.87 [0.81-0.93]) and lower all-cause mortality (HR 0.88 [0.83-0.93]).^38^ However, eGFR-stratified estimates for eGFR < 30 mL/min/1.73 m^2^ were not consistently available, leaving substantial uncertainty below this range and for de novo initiation in CKD G5.^38^ Safety signals were broadly consistent with known class effects (no clear difference in serious adverse events), but discontinuation owing to adverse events was higher (risk ratio 1.51 [1.18-1.94]); in CKD G4-G5, such tolerability-driven discontinuation may have outsized downstream consequences.^38^

### Real-World Data, Including Causal Inference–Oriented Observational Analyses

Large database studies evaluated GLP-1 receptor agonist use in advanced populations underrepresented in randomized trials. A key strength was size; a key limitation was residual confounding, particularly based on frailty, nutritional status, illness severity, and treatment selection.

Using the TriNetX platform (69 US healthcare organizations; 2018-2022), a propensity score–matched cohort study among adults with type 2 diabetes and CKD (eGFR ≤ 45 mL/min/1.73 m^2^) reported that GLP-1 receptor agonist use (vs nonuse) was associated with lower risks of dialysis initiation (HR 0.89 [0.85-0.93]), major adverse cardiovascular endpoints (HR 0.92 [0.88-0.95]), and all-cause mortality (HR 0.81 [0.78-0.84]).^39^

In a Taiwan nationwide cohort restricted to eGFR < 30 mL/min/1.73 m^2^, GLP-1 receptor agonists (vs dipeptidyl peptidase-4 inhibitors) were associated with a lower composite kidney outcome (subdistribution HR 0.72 [0.56-0.93]), whereas cardiovascular outcomes were not clearly improved, highlighting the importance of endpoint selection and residual confounding in advanced CKD.^40^

In adults with CKD G5 or kidney failure receiving dialysis in Taiwan, GLP-1 receptor agonist use (vs dipeptidyl peptidase-4 inhibitor use) was associated with lower all-cause mortality (HR 0.79 [0.63-0.98]) and lower sepsis- and infection-related mortality (HR 0.61 [0.40-0.91]).^41^

Taken together, observational data are consistent with potential kidney and survival benefits in advanced CKD strata while emphasizing cautious interpretation given residual confounding and incomplete capture of adjudicated kidney endpoints.

### Clinical Decision Points in CKD G4-G5: Initiate/Continue/Withhold/Discontinue/Reinitiate

Table 1^1^^,^^2^^,^^4^^,^^8^, ^9^, ^10^, ^11^, ^12^, ^13^, ^14^, ^15^, ^16^, ^17^, ^18^, ^19^, ^20^, ^21^, ^22^, ^23^, ^24^, ^25^, ^26^, ^27^, ^28^, ^29^, ^30^, ^31^, ^32^, ^33^, ^34^, ^35^, ^36^, ^37^, ^38^, ^39^, ^40^, ^41^ displays the clinical decision points in CKD G4-G5.

## Conclusions: Mission Possible With Monitoring

Across all the 4 pillars, the dominant implementation failure in CKD G4-G5 is often not absolute contraindication but avoidable loss of indicated therapies—missing pillars are not initiated, temporary interruptions become permanent discontinuations, and therapies are not reinitiated once acute triggers resolve. This matters because CKD G4-G5 confers the highest absolute kidney and cardiovascular risk and, therefore, potentially the greatest absolute benefit from multimechanistic risk reduction.^1^, ^2^, ^3^, ^4^, ^5^

More broadly, recent proposals in type 2 diabetes and CKD have emphasized earlier and more coordinated use of the 4 pillars rather than prolonged stepwise delay.^43^ Cross-trial actuarial analyses have also suggested that, in patients with type 2 diabetes and CKD, combining SGLT2 inhibitors, the nonsteroidal MRA finerenone, and GLP-1 receptor agonists on a background of RAS inhibition may yield substantial projected gains in kidney, cardiovascular, and overall survival.^44^ However, in CKD G4-G5, combination therapy should not be viewed as a rigid rapid-sequencing algorithm. Instead, the practical goal, as summarized in Fig 2, is to identify missing pillars, preserve tolerated therapies, and expand complementary therapy stepwise as potassium, kidney function trajectory, volume status, frailty, and monitoring feasibility allow. Evidence supporting accelerated or combination implementation remains derived largely from broader CKD populations rather than adults with advanced CKD specifically.

**Figure 2:**
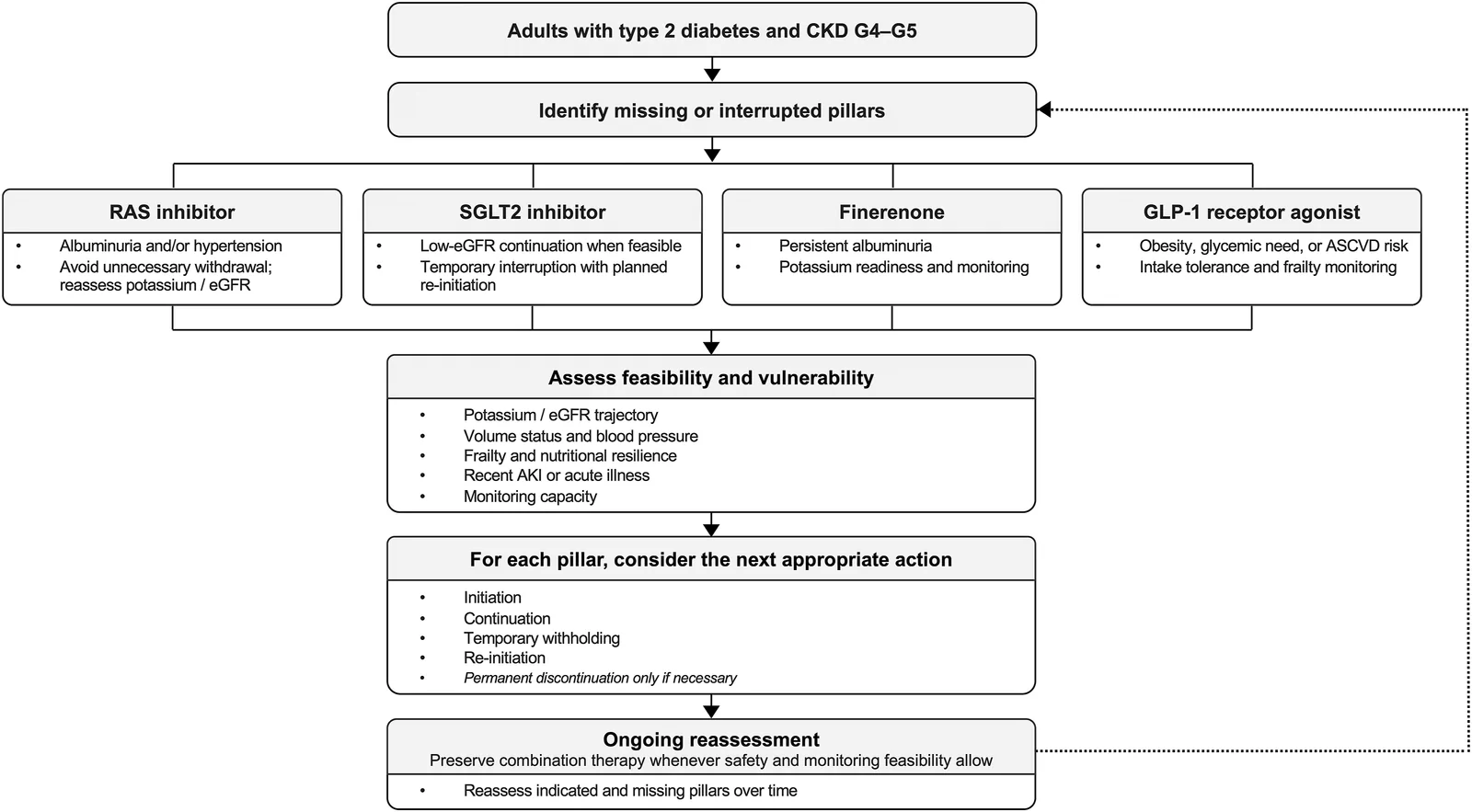
A practical approach to 4-pillar therapy in adults with type 2 diabetes and CKD G4-G5. The emphasis is not on following a rigid sequence of drug introduction, but on identifying missing or interrupted pillars, assessing clinical feasibility and vulnerability, and minimizing avoidable pillar loss over time. Across all pillars, temporary withholding during acute illness should be distinguished from permanent discontinuation and should generally be paired with reassessment and reinitiation when clinically appropriate. In CKD G5, many treatment decisions remain individualized and explicitly extrapolative because direct randomized evidence is limited or absent. Abbreviations: AKI, acute kidney injury; ASCVD, atherosclerotic cardiovascular disease; CKD, chronic kidney disease; eGFR, estimated glomerular filtration rate; GLP-1, glucagon-like peptide 1; RAS, renin-angiotensin system; Scr, serum creatinine; SGLT2, sodium-glucose cotransporter 2.

Current evidence supports a pragmatic, monitoring-first approach in CKD G4 and a more individualized, explicitly extrapolative approach in CKD G5.^1^^,^^2^ SGLT2 inhibitors have consistent kidney-protective signals down to eGFR 20-30 mL/min/1.73 m^2^ in randomized evidence and meta-analyses, arguing against automatic discontinuation based on eGFR thresholds alone.^15^, ^16^, ^17^^,^^19^^,^^21^, ^22^, ^23^ Finerenone shows robust albuminuria reduction and attenuation of chronic eGFR decline in CKD G4 under protocolized potassium monitoring, whereas de novo initiation in CKD G5 remains an evidence gap.^29^ GLP-1 receptor agonists now have dedicated kidney-outcome evidence anchored at eGFR ≥ 25 mL/min/1.73 m^2^, but tolerability-driven harms (reduced intake, dehydration, and frailty/sarcopenia) may have outsized consequences in advanced CKD and require proactive monitoring and dose titration.^34^

In practice, making 4-pillar therapy “mission possible” in CKD G4-G5 hinges on the following 3 principles: (1) explicit plans for laboratory monitoring and sick-day rules; (2) clear differentiation between temporary withholding and permanent discontinuation; and (3) deliberate, protocol-like reinitiation after acute complications resolve. Sequencing should be individualized, but the central practical goal is not to preserve a rigid order of drug introduction; rather, it is to minimize avoidable pillar loss and to maintain combination therapy whenever safety and monitoring feasibility allow. Future priorities include randomized or robust emulated-trial evidence for de novo initiation strategies in CKD G5, optimal sequencing and combination implementation in advanced CKD, and scalable monitoring models to prevent avoidable pillar loss in routine care.
